# Anterior segment biomechanics and intraocular pressure in microgravity: implications for future spaceflight studies

**DOI:** 10.1038/s41526-026-00592-2

**Published:** 2026-03-30

**Authors:** Alex Weaver, Joshua Ong, Baltaj S. Sandhur, Ritu Sampige, Alexander Black, Andrew G. Lee, John Berdahl, C. Robert Gibson, Thomas H. Mader

**Affiliations:** 1 Department of Ophthalmology, University of Florida College of Medicine, Jacksonville, FL USA; 2 Michigan Medicine, University of Michigan, Ann Arbor, MI USA; 3 Jacobs School of Medicine and Biomedical Sciences, Buffalo, NY USA; 4 Baylor College of Medicine, Houston, TX USA; 5 Center for Space Medicine, Baylor College of Medicine, Houston, TX USA; 6 Department of Ophthalmology, Blanton Eye Institute, Houston Methodist Hospital, Houston, TX USA; 7 The Houston Methodist Research Institute, Houston Methodist Hospital, Houston, TX USA; 8 Departments of Ophthalmology, Neurology, and Neurosurgery, Weill Cornell Medicine, New York, NY USA; 9 Department of Ophthalmology, University of Texas Medical Branch, Galveston, TX USA; 10 University of Texas MD Anderson Cancer Center, Houston, TX USA; 11 Texas A&M College of Medicine, Bryan, TX USA; 12 Department of Ophthalmology, The University of Iowa Hospitals and Clinics, Iowa City, IA USA; 13 Vance Thompson Vision, Sioux Falls, SD USA; 14 NASA Johnson Space Center, Houston, TX USA; 15 South Shore Eye Center, League City, TX USA; 16 NASA Ophthalmology, Houston, TX USA

**Keywords:** Biophysics, Diseases, Engineering, Medical research

## Abstract

Spaceflight acutely but transiently elevates intraocular pressure (IOP), often attributed to cephalad fluid shift and choroidal expansion. We propose that anterior segment mechanics, including lens–iris diaphragm position and conventional outflow loading, may contribute to early IOP changes. Comparing phakic and pseudophakic eyes, paired with anterior segment OCT and complementary imaging aboard the International Space Station, could define mechanisms and inform astronaut screening and ocular risk mitigation.

The effect of microgravity on human physiology has long intrigued scientists in the age of space exploration. Understanding the physiologic changes in the eyes induced by spaceflight has continued to be an elusive problem. Acceleration of both governmental and private space missions in recent years necessitates deeper understanding of spaceflight-associated physiologic and pathologic changes to improve long-term mission safety and outcomes.

Among the earliest and most consistently observed physiological changes in the eye during spaceflight is a temporary increase in intraocular pressure (IOP). The first study to identify an elevated IOP in this setting was achieved by Draeger on the Spacelab D-1 mission, with an IOP increase of 20–25% from baseline at minute 44 of microgravity exposure^1^. A later Draeger study identified an elevation of IOP by 92% during initial exposure to microgravity, with an earliest measured increase at sixteen minutes into microgravity exposure, suggesting that the readings on Spacelab D-1 were already skewed by an “adaptation process.”^2^ These early in-flight measurements should be interpreted with caution, as the Ocuton S tonometer used in the Draeger studies has demonstrated limited agreement with Goldmann applanation tonometry in comparative evaluations (with only approximately one-third of readings within ±2 mmHg)^3^. Nonetheless, the rapid timing and consistent directionality of IOP elevation shortly after microgravity exposure remain physiologically informative.

Much of the current literature proposes that the IOP elevation is due to cephalad fluid shifts under microgravitational conditions^2,4–6^. These fluid shifts are thought to stimulate sudden choroidal expansion leading to an acute rise in IOP. Alternative mechanisms, however, include increased aqueous humor production through enhanced ciliary body perfusion and restricted outflow due to episcleral venous congestion (Fig. 1). However, there has been limited discussion of the natural crystalline lens position and its effect on IOP in a microgravitational environment. Some authors have theorized that subtle, gravity-mediated changes in lens position may contribute to early-phase IOP elevation in microgravity by indirectly narrowing the iridocorneal angle and impeding aqueous outflow via alteration of the zonular traction on the ciliary body and uveal tract ^7^.

**Fig. 1:**
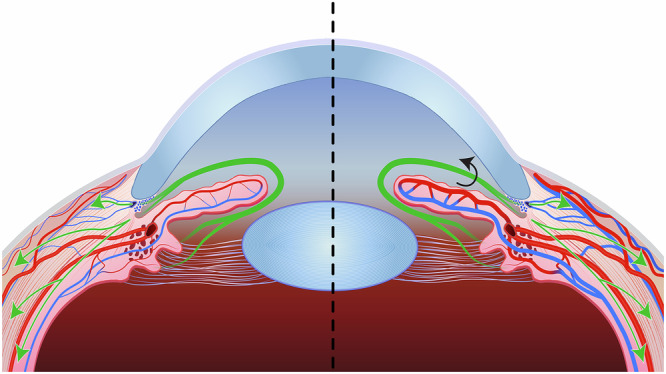
Left side of image showing normal terrestrial aqueous humor production and drainage. Right side of image showing engorged vessels simulating a simplified cephalad fluid shift in microgravity.

This hypothesis, though untested under spaceflight conditions, could be supported by terrestrial studies examining postural shifts and lens-related angle dynamics. As spaceflight becomes more accessible to a broader and older population, including individuals with pseudophakia and even glaucoma, it is increasingly important to explore multifactorial contributors to IOP changes in concert with the more established explanations.

The biophysics of a natural crystalline lens in microgravity continues to be debated and studied. Some authors dispute the impact that terrestrial gravity has on an accommodating lens, citing experimental error^8^. However, studies by Lister et al. provide a basis for considering how lens position may change under altered gravitational forces. Using prone posturing paired with varying levels of pharmacologically induced accommodation, they identified a “small but real” decrease in anterior chamber depth (ACD) ranging from 0.04 mm to 0.12 mm as accommodation increased^9^. Their explanation for this change aligns with the widely accepted Helmholtz theory of zonular laxity due to contraction of the ciliary muscle during active accommodation (near-sight), which views the crystalline lens as a dynamic structure, where accommodation induces predictable changes in lens curvature and position ^10^.

Extrapolating from these terrestrial findings, it is plausible that exposure to microgravity could promote a passive anterior shift of the lens-iris diaphragm, resulting in further reduction of anterior chamber depth (ACD) and narrowing of the trabecular meshwork outflow tract. While this study does not connect these changes to IOP, their findings raise the question of whether shifts in lens position influence iridocorneal angle configuration and outflow dynamics. Such shifts could be accommodation related, driven by ciliary muscle contraction and relaxation with corresponding changes in zonular tension and lens elasticity. They could also be passive, resulting from altered pressure and volume forces in microgravity. Interestingly, Wang et al. observed anterior displacement of the lens with narrowing of the angle opening distance (AOD) during supine positioning, which would suggest that gravity itself may not be a major direct effector of lens-angle dynamics^11^. The authors proposed that in terrestrial cases, increased vascular stasis in supine positioning leads to an increased choroidal volume, which transmits an anterior force via the vitreous to the lens and, indirectly, the iridocorneal angle. Additionally, early terrestrial head-down tilt-testing as a model of microgravity hypothesized similar choroidal engorgement, citing Smith and Lewis’ experiment showing a 20 mmHg IOP increase with a 20 μL increase in choroidal volume^12,13^. The role of choroid expansion was also discussed as a possible factor in the original Spaceflight Associated Neuro-Ocular Syndrome (SANS) paper by Mader et al.^14^. Further, Macias et al. identified a sustained increase in peri-papillary choroidal thickness by around 2.8% with a paired decrease in anterior chamber depth^15^. Thus, much of the existing literature studying IOP fluctuations in microgravity has focused on the role of choroidal expansion and impediment of aqueous humor outflow resistance that may occur.

A plausible complementary pathway that may influence uveal fluid balance is lymphatic drainage from the anterior uvea. Lymphatic marker positive channels have been described in the human ciliary body, supporting an anatomic basis for a uveolymphatic outflow^16^. Functional studies further support drainage from the eye to cervical lymphatic networks, demonstrated by tracer detection in cervical lymph nodes and recovery via cannulated cervical lymphatic pathways^17,18^. In parallel, the cervical outflow environment is altered in spaceflight and microgravity analogs, with retrograde internal jugular venous flow reported during ISS missions and reduced deep cervical lymphatic drainage observed during head-down tilt, consistent with prior discussion that altered gravity may disadvantage cranial and cervical lymphatic clearance ^19–21^.

If cervical lymphatic clearance is relatively constrained during microgravity adaptation, excess interstitial fluid could be retained within uveal tissues, creating a capacitance or reservoir effect in the choroid and potentially the ciliary body. A downstream pressure environment could then matter for IOP if it increases the effective resistance that aqueous must traverse along the conventional pathway. In this setting, the conventional outflow system may behave nonlinearly, because valve-like structures have been described at Schlemm canal inlets and at collector channel outlets, creating a pressure-sensitive gating mechanism that may be influenced by small changes in pressure gradients and tissue apposition^22^. In addition, Schlemm’s canal has been characterized as a lymphatic-like vessel that is responsive to lymphangiogenic signaling and requires lymphatic regulators for its identity and integrity, supporting the concept that conventional outflow shares molecular features with lymphatic-associated biology^23,24^. Together, these concepts provide a framework in which uveal fluid retention and pressure shifts in microgravity could interact with valve-like conventional outflow behavior to transiently reduce outflow facility, while remaining complementary to blood vessel driven fluid shift mechanisms.

Taken together, the mechanisms discussed above suggest that microgravity may influence IOP through coupled effects on uveal volume handling and aqueous outflow resistance. Within this broader context, two anatomic processes, choroidal expansion and anterior displacement of the lens–iris diaphragm, may be particularly relevant to anterior segment configuration and thus outflow dynamics. Building upon this foundation, we broaden the discussion to consider the potential contribution of crystalline lens position to spaceflight-associated IOP behavior.

## Phakic vs pseudophakic rationale and existing evidence

To isolate the natural lens’s possible contribution to IOP changes in microgravity, a useful model is to compare phakic eyes to pseudophakic eyes. Studies have shown that cataract surgery increases AOD and widens the iridocorneal angle, with an especially pronounced effect in eyes with shorter axial lengths^25^. Further, the Effectiveness of Early Lens Extraction for the Treatment of Primary Angle-Closure Glaucoma (EAGLE) trial demonstrated that clear-lens extraction reduces IOP in patients at risk of primary angle closure and primary angle closure glaucoma^26^. Additionally, Kim et al. provide supporting data, demonstrating increased AOD and ACD in pseudophakic patients compared to phakic patients^18^. These terrestrial data highlight how removal of the crystalline lens modifies anterior segment anatomy and outflow, providing a conceptual model for evaluating lens-related influences on IOP in microgravity. In microgravity analog testing, optical biometry obtained alongside tonometry has included lens thickness, which did not demonstrate a significant condition-dependent change despite an acute IOP increase, suggesting that lens thickening alone is unlikely to account for early IOP elevation^27^. To our knowledge, published studies remain limited that dynamically track lens-related anterior segment parameters during the earliest phase of microgravity exposure. As a result, relationships between time-dependent lens configuration changes, such as lens vault and iridocorneal angle narrowing, and early IOP behavior remain largely uncharacterized, supporting the need for the in-flight anterior segment imaging approaches outlined below. This gap is most apparent in the spaceflight literature. Notably, only two pseudophakic astronauts have been discussed, and in both cases, IOP measurements began 30 days after launch, which may have missed the initial biophysical transition period into microgravity ^28,29^.

## Two-phase IOP hypothesis and mechanistic interpretation

While the pseudophakic model offers some insight into the lens’ influence on IOP, it does not capture the full complexity of spaceflight associated physiologic changes. Within the 2018 discussion by Mader et al., IOP measured at later stages (launch, 30d, 90d, and 140d) showed no variation from preflight readings, despite ultrasound-measured choroidal expansion^29^. This suggests a two-phase pattern to IOP changes in space; an acute spike followed by a period of adaptation^2,30^. The timing and magnitude of these phases remain poorly characterized, emphasizing the need for early in-flight measurement within the first hours to days of exposure. While choroidal expansion seems to be the most significant factor in this acute IOP elevation, further evaluation of factors involving the anterior segment, such as lens displacement, zonular laxity, angle narrowing, and mechanical changes in outflow facility, may contribute, in part, to this early phase rise in IOP. Within a pump-conduit framework of conventional outflow ciliary body, scleral spur, and trabecular meshwork, tension may alter pressure-dependent gating behavior. Johnstone et al. depict a “ball valve” effect in which chamber deepening increases ciliary body, scleral spur, and trabecular meshwork tension, whereas iridectomy eliminates chamber deepening and the associated tension, suggesting that anterior segment configuration itself can meaningfully change outflow facility^22^. Microgravity associated anterior lens-iris diaphragm shifts could therefore interact with this tension-dependent regulation, potentially reducing the protective effect of chamber deepening and contributing to early-phase outflow impairment. Later-stage IOP tends to return toward baseline despite persistent cephalad fluid shift, suggesting physiologic adaptation in the vascular and downstream outflow environment ^21,31^.

## Imaging recommendations and ISS capabilities

**Fig. 2:**
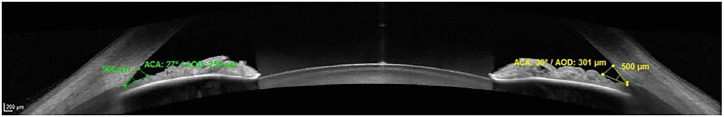
Anterior segment optical coherence tomography (AS-OCT) demonstrating anterior chamber angles (ACA) and angle opening distance (AOD). Image reprinted from (Asam et al.^33^) under a Creative Commons License.

To further explore these facets of spaceflight-associated physiological changes, high-resolution imaging of the anterior segment before, during, and after exposure to microgravity may be required. Current literature has mainly focused on microgravity’s effects on the posterior segment, largely in the study of SANS. Recent work has emphasized the relevance anterior segment imaging may provide during spaceflight. Sandhur et al. focuses on implications and operational feasibility for the use of anterior segment imaging, advocating for its routine use to characterize changes^32^. There still remains a gap in knowledge regarding the potential influence of the anterior segment and angle dynamics. This discrepancy further underscores the importance for utilization of imaging techniques to help address this gap. Targeted imaging of anterior segment structures could clarify the mechanisms driving early intraocular pressure changes observed in spaceflight. The ISS currently has the capacity to perform OCT imaging (Heidelberg Spectralis OCT2) on board. This technology has been used to image the posterior segment of the eye since 2013. With the addition of an add-on anterior segment lens, the Spectralis can be easily adapted for anterior segment OCT (AS-OCT). The disadvantage of AS-OCT compared to other imaging modalities such as ultrasound biomicroscopy is the inability to view structures posterior to the iris, however angle dynamics could be studied in high-resolution with AS-OCT. Further, the predefined screening patterns, automated alignment features, and guidance prompts can improve ease of use and aid in the acquisition of consistent images of the anterior segment^33^. Consistent use of AS-OCT would allow longitudinal monitoring of the various parameters discussed and would be useful for tracking changes to the lens positioning such as the lens vault, ACD, AOD, and anterior chamber angles during short and long-term spaceflight^33,34^ (Fig. 2).

Anterior segment ultrasound biomicroscopy (AS-UBM) could serve as a complementary modality to AS-OCT by enabling direct visualization of structures posterior to the iris, including the ciliary body and supraciliary space, which are central to several proposed mechanisms of microgravity-associated outflow change^35^. If microgravity disadvantages uveal fluid clearance and promotes an interstitial reservoir, AS-UBM could help assess whether this manifests as ciliary body configuration change, ciliary body thickening, anterior rotation, or supraciliary fluid, all of which are established UBM-detectable patterns that can narrow the iridocorneal angle and elevate IOP^36,37^. Ultrasound capability has already been used aboard the ISS for comprehensive ocular examination using B- and M-mode imaging with remote expert guidance, suggesting a potential operational pathway for adding targeted anterior segment ultrasound protocols^38,39^. However, implementation of high-frequency AS-UBM in microgravity would likely require dedicated anterior segment probes and a sealed coupling interface to contain the immersion medium to enable safe, reproducible acquisition ^40^.

Beyond structural imaging, monitoring that captures ocular pulsatility may further contextualize early versus adapted IOP behavior in microgravity. The PASCAL dynamic contour tonometer (DCT) provides both IOP and ocular pulse amplitude (OPA), with OPA defined as the difference between systolic and diastolic IOP and representing the pulsatile wave produced by cyclic changes in intraocular blood volume during the cardiac cycle^41,42^. As a complementary readout to AS-OCT and AS-UBM, OPA could help characterize whether acute IOP elevations occur with concurrent changes in ocular pulsatility consistent with altered vascular or distal outflow loading, while also offering a potential marker of adaptation over time. Notably, DCT has been used to measure IOP and OPA in crew members following long-duration ISS missions, supporting feasibility of incorporating OPA as an outcome metric in the spaceflight context^43^. Systemic factors, including blood pressure, vascular perfusion, and venous hemodynamics, may influence the magnitude of ocular biomechanical responses and should be captured as covariates in future studies ^19,44^.

Currently, one of the most commonly studied in vivo biomechanical domains in anterior segment research is corneal biomechanics. Commercially available platforms include the Ocular Response Analyzer (ORA) and Corneal Visualization Scheimpflug Technology (Corvis ST). Both use a controlled air pulse with optical detection of the corneal response to derive biomechanical parameters^45–47^. The ORA provides measures such as corneal hysteresis and corneal resistance factor, while the Corvis ST reports deformation-based parameters related to corneal stiffness and dynamic response^46^. Corneal hysteresis correlates with IOP, although the association is modest^47,48^. In principle, these tools could help characterize biomechanical contributors to microgravity-associated IOP behavior, either in spaceflight or in terrestrial models of microgravity. However, implementation in spaceflight would likely require validation and operational adaptation, with particular attention to stable positioning and repeatable alignment to support reproducible acquisition^46,47^.

We propose to understand the potential short and long term consequences of space flight on ocular health in the burgeoning era of commercial space flight, alongside ongoing governmental work and expansion of military space presence. Further, we can elucidate a deeper understanding of terrestrial models by creating a reliable comparative model and potentially improve astronaut screening and safety parameters. During the era of spaceflight, many studies have examined physiological changes induced by exposure to a microgravitational environment, including potentially serious elevations of IOP. However, the anterior segment’s contribution to these IOP changes remains underexplored. Based on the current knowledge and understanding of this phenomenon, we propose that the potential contribution of the anterior segment warrants further in-vivo analysis under appropriate testing conditions. This includes detailed examination of the lens-iris diaphragm, which may bow forward under microgravitational forces, causing decreased AC depth, narrowing of the iridocorneal angle, and obstruction of aqueous outflow. Combining this theory with existing propositions of choroidal volume expansion and cephalad fluid shifts allows for a fluid multifactorial understanding of the IOP spikes, which have been documented in prior studies. Integrating anterior segment OCT, AS-UBM, novel approaches to tonometry, and corneal biomechanical instrumentation into ISS ophthalmic study would enable in-vivo quantification of lens and angle dynamics during microgravity adaptation.

**Fig. 3:**
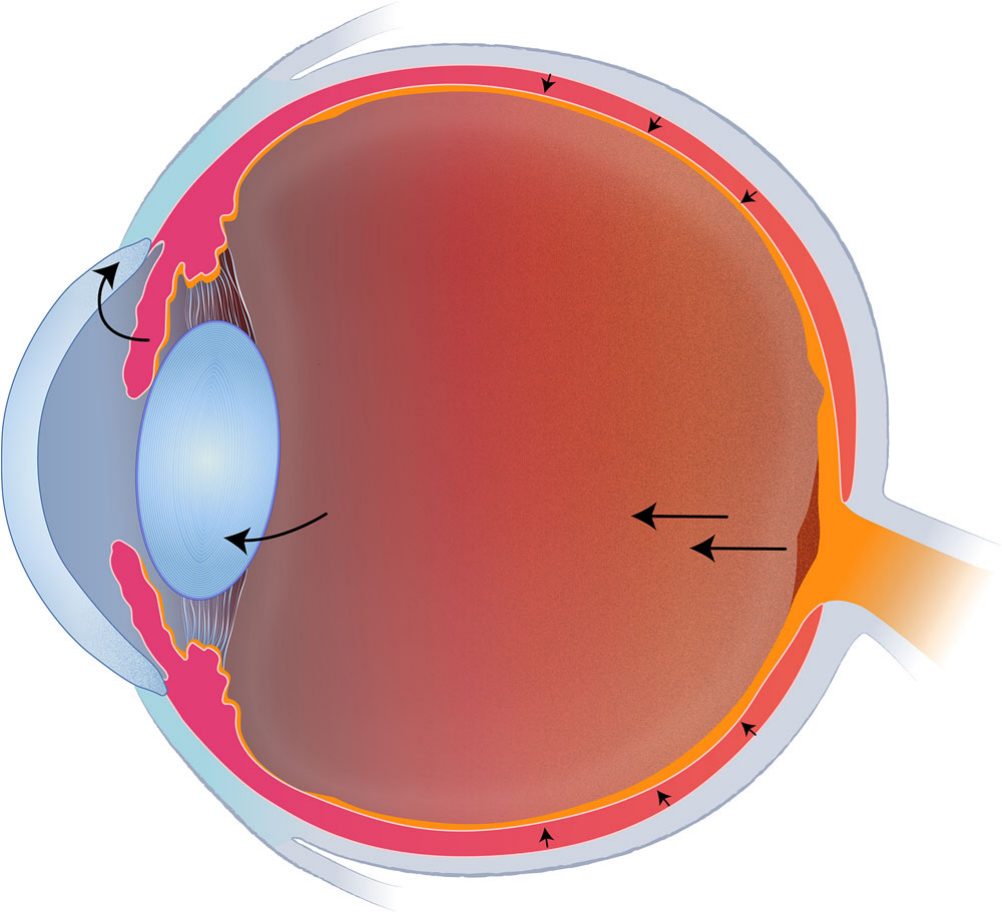
Model of the eye in a microgravity environment, highlighting choroidal expansion (right side of photo), transmitting force via the vitreous humor to the mobile crystalline lens.

## Data Availability

No datasets were generated or analysed during the current study.
